# PFAS Degradation in Ultrapure and Groundwater Using Non-Thermal Plasma

**DOI:** 10.3390/molecules26040924

**Published:** 2021-02-09

**Authors:** Davide Palma, Dimitra Papagiannaki, Manuel Lai, Rita Binetti, Mohamad Sleiman, Marco Minella, Claire Richard

**Affiliations:** 1 Université Clermont Auvergne, CNRS, Sigma Clermont, ICCF, 63178 Aubière, France; davide.palma@etu.uca.fr (D.P.); mohamad.sleiman@sigma-clermont.fr (M.S.); 2 SMAT S.p.A., Research Centre, C.so Unità d’Italia 235/3, 10127 Torino, Italy; dimitra.papagiannaki@smatorino.it (D.P.); rita.binetti@smatorino.it (R.B.); 3 IRIS s.r.l., Via Papa Giovanni Paolo Secondo 26, 10043 Orbassano, Italy; manuel.lai@irissrl.org; 4 Department of Chemistry and Interdepartmental Centre Nanostructured Interfaces and Surfaces (NIS), University of Torino, Via Pietro Giuria 5, 10125 Torino, Italy

**Keywords:** non-thermal plasma, PFAS, NO_x_, water treatment, advanced oxidation processes

## Abstract

Perfluoroalkyl substances (PFAS) represent one of the most recalcitrant class of compounds of emerging concern and their removal from water is a challenging goal. In this study, we investigated the removal efficiency of three selected PFAS from water, namely, perfluorooctanoic acid (PFOA), perfluorohexanoic acid (PFHxA) and pefluorooctanesulfonic acid (PFOS) using a custom-built non-thermal plasma generator. A modified full factorial design (with 2 levels, 3 variables and the central point in which both quadratic terms and interactions between couple of variables were considered) was used to investigate the effect of plasma discharge frequency, distance between the electrodes and water conductivity on treatment efficiency. Then, the plasma treatment running on optimized conditions was used to degrade PFAS at ppb level both individually and in mixture, in ultrapure and groundwater matrices. PFOS 1 ppb exhibited the best degradation reaching complete removal after 30 min of treatment in both water matrices (first order rate constant 0.107 min^−1^ in ultrapure water and 0.0633 min^−1^ in groundwater), while the degradation rate of PFOA and PFHxA was slower of around 65% and 83%, respectively. During plasma treatment, the production of reactive species in the liquid phase (hydroxyl radical, hydrogen peroxide) and in the gas phase (ozone, NO_x_) was investigated. Particular attention was dedicated to the nitrogen balance in solution where, following to NO_x_ hydrolysis, total nitrogen (TN) was accumulated at the rate of up to 40 mg^N^ L^−1^ h^−1^.

## 1. Introduction

The availability of clean water sources is worldwide rapidly diminishing, so the availability of effective technologies capable to ensure water reuse has become a primary necessity. This is not only a challenge for those countries having scarce freshwater resources but also for densely populated regions like Europe where over 64% of freshwater is collected from rivers [1]. Most operating wastewater treatment plants (WWTPs) rely on traditional treatments (e.g., biological treatment, sedimentation and active carbon filtration) unable to totally remove a large number of bio-recalcitrant anthropogenic compounds often referred to as CECs (Contaminants of Emerging Concern) that are returned to the environment [2,3,4]. For most CECs typical surface water concentration ranges from few ppt to tens of ppb but removal efficiency can drastically change depending on pollutant’s initial concentration, wastewater characteristics and the type of adopted technology [5,6]. The presence of CECs in water bodies not only limits water reuse but it is also deleterious for the aquatic fauna as many CECs are toxic compounds and often endocrine disruptors [7,8]. Among CECs, perfluoroalkyl compounds (PFAS) represent one of the most problematic class of compounds due to their resistance to conventional water treatments and persistence in the environment [9,10,11]. 

Advanced Oxidation Processes (AOPs) have been proposed to be implemented together with the conventional biological treatments to efficiently address the removal of CECs from wastewater [12,13,14,15]. These processes rely on chemical and/or physical processes generating highly reactive radical species (such as HO^●^ and SO_4_^●−^) able to react non selectively with the most organic compounds in solution, including, bio-recalcitrant compounds [16,17]. Among the most conventional AOPs are the Fenton and photo-Fenton processes, ozonation, UV photolysis, H_2_O_2_ and heterogeneous photocatalysis [18,19]. While ozonation and UV photolysis are relatively simple processes that have also been proven effective in degrading CECs, their large-scale application has been strongly limited by high operational costs.

Water plasma is attracting today the attention of a growing number of researchers due to its promising performances in water treatment applications [20]. Non-thermal plasmas in particular have been intensively studied due to a series of practical advantages like low energy consumption and simple operative equipment [21,22]. From a chemical standpoint, the main advantage in using such technology is the ability to activate different processes at the same time such as the formation of highly reactive species like H^●^, O, HO^●^ radicals, free electrons and ozone, as well as oxidants (e.g., hydrogen peroxide), the generation of ultraviolet light due to the plasma discharge light emission and the generation of shockwaves and high-density electric fields. The degradation mechanism of pollutants is therefore complex. Different electrodes configuration has been explored but the most used geometry for water plasma application is the so called point-to-plane or needle-to-plane geometry where positive or negative polarity is applied to a needle-like electrodes while a larger plate-shaped electrode is grounded [22,23,24]. According to the polarity applied to the electrode, important differences in terms of treatment efficiency can be observed [21,25]. Due to the chemical stability of the C-F bond in PFAS, these compounds are extremely refractory to degradation resulting in insufficient abatement when using traditional water treatment technologies. Recently, the use of non-thermal plasma for PFAS removal has been investigated by several research groups. Despite the promising results obtained so far, an exhaustive comprehension of the degradation mechanism and the study of simultaneous treatment of different PFAS in real water matrices is poorly documented in the literature [26,27]. 

The aim of this work was to optimize the performances of a custom-made high voltage discharge generator (European Patent n EP 3 023 392 B1) for water treatment applications and then, to test the removal efficiency of the optimized plasma system on three perfluoroalkyl compounds (PFAS). In the first part of the work, a modified full factorial design (with 2 levels, 3 variables and the central point in which both quadratic terms and interactions between couple of variables were considered) was used to maximize water pollutants removal and the discoloration of methylene blue solution was used as response. Then, the generation of reactive species like hydrogen peroxide, ozone and NO_x_ has been evaluated during plasma treatment. Attention was dedicated to the nitrogen balance in the solution. Last, the removal efficiency of the plasma treatment on three recalcitrant PFAS (perfluorooctanoic acid (PFOA), perfluorohexanoic acid (PFHxA) and pefluorooctanesulfonic acid (PFOS)) was investigated individually and in mixture, both in ultrapure water and in a real groundwater matrix. Compared to other systems, this discharge generator is capable of delivering high voltage pulses that rise from zero to 120 kV in five nanoseconds. This fast-rising potential is a crucial feature capable of ensuring the generation of the streamer even when working with highly conductive water matrices. Each pulse consists of multiple peaks of 10 nanoseconds reaching 90 to 120 kV. The total duration of the pulse is approximately 250 nanoseconds, considerably shorter than standard Pulsed Electric Field (PEF) systems. 

## 2. Materials and Methods

### 2.1. Chemicals

PFOA (Perfluorooctanoic acid, analytical standard), PFOS (Perfluorooctanesulfonic acid, solution 40% *w*/*w*), PFHxA (perfluorohexanoic acid, analytical standard), methylene blue (MB, purity > 95%), methyl orange (MO, purity 85%), furfuryl alcohol (FFA, purity 98%), sodium nitrite (purity ≥ 97%), sodium nitrate (purity ≥ 99%), ammonium chloride (purity ≥ 99.5%), potassium hydrogen phthalate (purity ≥ 99.5%), sodium hydroxide (purity ≥ 98.0%), ammonium heptamolybdate tetrahydrate (purity ≥ 99.0%), potassium iodide (purity ≥ 99.0%) and hydrogen peroxide (solution 30% *w*/*w*) were purchased from Sigma-Aldrich and used without further purification. Dry natural organic matter was purchased from International Humic Substances Society (product code 2R101N). 2-Propanol and chromatographic UHPLC-grade methanol were purchased from Carlo Erba Reagents. All aqueous solutions, if not differently specified, were prepared using ultrapure water Millipore Milli-Q™ (TOC < 2 ppb, 18.2 MΩ cm). 

### 2.2. Plasma Generator

**Figure 1:**
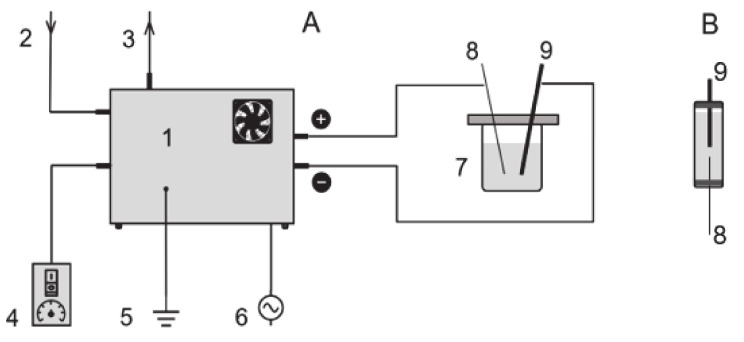
Schematic representation of the plasma generator connected to the Pyrex glass (PG) reactor (**A**): (1) plasma generator, (2) air inlet, (3) air outlet, (4) plasma switch and frequency control, (5) ground, (6) 220 V power supply, (7) PG reactor, (8) sintered tungsten electrode, (9) stainless steel electrode. polypropylene (PP) reactor (**B**).

The high voltage discharges were generated with a custom-built Marx generator powered with 220 V AC equipped with a pulse-width modulation circuit, a high-voltage transformer and four 990 pF capacitors. A small continuous flow of compressed air with relative humidity around 14% was fed to the spark-gaps’ chamber to stabilize the generator’s internal atmosphere. The discharge’s peak voltage was typically 100–130 kV with peak current values of 20–40 A. The pulse duration was approximately 250 ns and the frequency of discharge could be manually adjusted between 5 and 17 Hz. Electrical measures have been performed using a BK Precision 2190D oscilloscope. Total absorbed power of the generator laid between 299 and 322 W. Water was treated using two different reactors: a 20 mL cylindrical polypropylene (PP) reactor was used in the first part of the work aimed to the optimization of working parameters while a 50 mL Pyrex glass (PG) reactor was used for all other experiments. The PP reactor on one site limited the risk of damage of the reactor wall as a consequence of the generated shock waves, whilst on the other side caused Total Organic Carbon (TOC) and Total Nitrogen (TN) potential contamination from plastics and rubber parts. For both reactors, the same electrodes have been used consisting in one stainless steel bar having 10 mm diameter and a tungsten sintered electrode with a diameter of 3 mm on the other side. In the PG reactor the distance between the electrodes determined whether the discharge was forming underwater (distance < 2 mm) or at the water surface (distance > 2 mm). For both reactors, the headspace was in connection with the room air. Schematic representation of the experimental setup is shown in Figure 1. Both the plasma generator and the reactors were positioned inside a Faraday cage.

### 2.3. Analytical Methods

PFAS analyses were carried out using a SCIEX X500R QTOF system coupled to a Shimadzu ExionLC UHPLC system equipped with a Luna^®®^ Omega Polar C18 100 LC Column (3 µm particle size, 100 × 2.1 mm) heated at 40 °C. Injection volume was 50 µL and the mobile phase was a mixture of 5 mM Ammonium Acetate in H_2_O (A) and 5 mM Ammonium Acetate in MeOH (B) at a 0.350 mL/min flow. The gradient elution started with 95% A and 5% B, which was held up to 1 min and then gradually changed up to 100% B within 10 min. This ratio was kept for 2 min and then gradually reversed into the initial conditions until 15 min of elution. MS analysis was performed with the X500R QTOF mass spectrometer, which operated in negative Electrospray Ionization mode (ESI). The source conditions were set as the following: gas temperature 500 °C, Curtain gas pressure 30 psi, ion spray voltage −4500 V. The acquisition was done using the SWATH (Sequential Window Acquisition of All Theoretical Fragment Ions) mode, which is a Data Independent Acquisition (DIA) method where all the precursor ions within the determined *m*/*z* range are fragmented in a methodical and unbiased way. The SWATH mode consisted of a TOF MS full scan, followed by MS/MS experiments with fixed quadrupole (Q1) isolation windows. The full scan covered a mass range of *m*/*z* 100–1500 with an accumulation time of 0.05 s. The Q1 precursor ions’ isolation strategy (MS/MS scans) covered a mass range of *m*/*z* 50–1500 with window width of 25 Da and each SWATH window had an accumulation time of 0.03 s. A collision energy (CE) of 35 eV with a CE spread of 15 eV was applied. The chromatograms and mass spectra were processed with the SCIEX OS 1.7 software. PFAS quantification was based on six-points calibration curves (10, 50, 100, 500, 1000 and 5000 ppt) having R^2^ > 0.99 for all compounds.

The concentration of NO, NO_2_, NO_x_ and O_3_ produced during plasma treatment was determined using the Horiba APOA-360 Ambient Ozone Monitor and the Horiba APNA-370 Ambient NO_x_ Monitor. Air was sampled 2 cm above treated water surface using a polytetrafluoroethylene (PTFE) gas sampling tube and the air flow was then split in two and sent to the NO_x_ and ozone analyzers simultaneously. Total sampling flow was 1.5 L/min.

Total nitrogen was measured using a Shimadzu TOC-VCSH Total Organic Carbon Analyzer, equipped with an ASI-V autosampler and fed with zerograde air. Main nitrogen inorganic ions (NO_3_^−^, NO_2_^−^ and NH_4_^+^) were determined with a Dionex DX 500 ion chromatograph equipped with a gradient pump GP40, an electrochemical suppression unit (ASRS 300 for anions and CERS 500 for cations), an ED40 detector and a Rheodyne injector (100 μL injection loop). The separation columns used were Dionex Ion Pac AS9-HC with an AG9-HC guard column for the anions and Dionex CS12A with a CG12A guard column for the cations. The used eluents were 9 mM K_2_CO_3_ (anions) and 20 mM methanesulfonic acid (cations). In both cases, the total flow rate was 1 mL/min. 

The formation of H_2_O_2_ along plasma treatment was determined spectrophotometrically at 350 nm in presence of iodide (method is reported in Supplementary Materials). 

All spectrophotometric analyses were performed using a Varian CARY 100 Scan double-beam UV–Vis spectrophotometer, using quartz cuvettes with 10 mm path length.

## 3. Results and Discussion

### 3.1. Plasma Parameters Optimization

As the plasma generator used in this work had never been tested for organic pollutants degradation, the first part of the experimental work was dedicated to the optimization of the following operational parameters: applied polarity, frequency of discharge, water conductivity, distance between the electrodes and reactor material. This first experimental part was carried out using the polypropylene (PP) reactor. 

#### 3.1.1. Applied Polarity

To understand the effect of polarity in our system, the degradation of Methyl Orange (MO) was performed using both positive and negative polarity applied to the pointy tungsten electrode. The test was performed on 15 mL of MO solutions at two different concentrations, 3 × 10^−5^ M and 5.5 × 10^−5^ M. Degradation tests of 15 min were performed with both polarities and in both cases the degradation profiles for MO were faster with positive polarity (at the two MO concentrations the slope was higher by 1.8 and 3.9-fold) (Figure S1).This behavior has been previously reported for similar setups and can be explained in terms of volume of solution covered by the discharge [21]. Higher rate of formation of HO^●^ for positive discharges have been reported as well [25].

#### 3.1.2. Water Conductivity, Frequency of Discharge and Distance between Electrodes

The development of the DOE was carried out to investigate the role of three factors on the degradation of methylene blue (MB) (full list of the experiments is reported in Table S1). The investigated variables and explored ranges are:

- frequency of the electrical discharge (5–17 Hz)
- distance between the electrodes (1–10 mm)
- water conductivity (20–300 µS/cm)

MB was selected because exhibited faster degradation rates compared to MO and gave a more reliable and faster feedback. The initial absorbance of the solution at 665 nm was fixed to 0.7 and discoloration was monitored measuring the absorbance at this wavelength (Abs_665nm_). All solutions were prepared in ultrapure water and conductivity was adjusted using sodium chloride. The DOE consisted of a series of 13 duplicated experiments performed in randomized order. The obtained regression equation (R^2^ for the model 95.6%) avoiding the not statistically relevant terms is:

k = 0.1883 + 0.03144 × a − 0.07976 × b + 0.001587 × c + 9.763 × 10^−4^ × a × b − 8.206 × 10^−5^ × a × c + 6.383 × 10^−3^ × b^2^ − 3.847 × 10^−6^ × c^2,^

where k is the exponential term of the fitting equation used for the degradation profiles of MB (Abs_665_ = y_0_ + A × 10^(−k t)), a is the frequency of discharge (Hz), b is the electrodes distance (mm) and c is water conductivity (µS/cm).

The frequency of discharge showed a positive correlation with the rate of discoloration of the MB solution (Table S2). This is intuitively explainable in term of higher production rate of reactive species. The water conductivity was negatively correlated with the rate of discoloration. This can be explained considering that as water conductivity increased, plasma discharge formation was limited by the competitive phenomenon of charge transport by ions in solution. Furthermore, at higher conductivities the length of the discharge shortened and the efficiency of reactive species production might decreased [28]. Considering the results of DOE, all following experiments have been performed in the Pyrex glass (PG) reactor in the optimized conditions, that is, with a frequency of discharge of 17 Hz, distance between electrodes of 5 mm and without addition of sodium chloride.

### 3.2. Identification of Reactive Species

#### 3.2.1. Role of HO^●^

**Figure 2:**
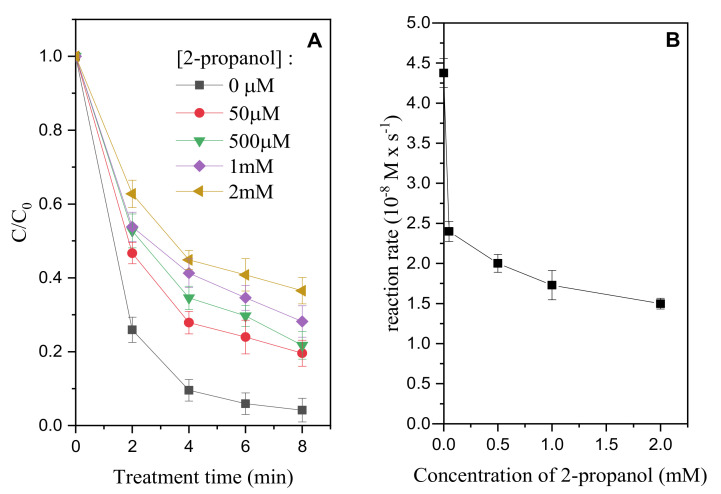
Degradation profiles of methylene blue (MB) (initial concentration 10^−5^ M) in the presence of increasing concentrations of 2-propanol, treated in the PG reactor (**A**). Evolution of the reaction rate for MB degradation in presence of increasing concentration of 2-propanol (0–2 mM range) (**B**).

To estimate the contribution of HO^●^ in the degradation process during plasma treatment, a solution of MB 10^−5^ M, was treated in the PG reactor, in the presence of increasing concentration of 2-propanol (0–2 mM). Figure 2 shows the degradation profile for MB in the presence of different concentration of 2-propanol and the transformation rate of the substrate with the increase of the concentration of the scavenger. The initial degradation rate of MB (computed as the product between the first order kinetic constant obtained from the exponential fit of the MB degradation profiles and the initial substrate concentration) was inhibited by 2-propanol up to a plateau value of ≈1.5 × 10^−8^ M s^−1^. The high inhibition of the MB transformation rate with the increase of 2-propanol suggested (i) a high production of hydroxyl radicals; (ii) an active role of this species during the degradation of MB in the explored experimental conditions and (iii) the presence of other processes concurring in the degradation of MB alternative to its reaction with HO^●^ (e.g., oxidation/reduction of the substrate at the electrodes, UV/Vis photolysis…).

#### 3.2.2. H_2_O_2_ Production

The formation of H_2_O_2_ is commonly observed in plasma treated solutions [28,29] through the reactions reported below (Equations (1)–(7)). The formation of H_2_O_2_ is initiated by the formation during the high energetic discharge of high-energy electrons (*e^−^) able to react both with dissolved oxygen (Equation (1)) and water (Equation (2)) [30].

O_2_ + *e^−^ → 2 O + *e^−^ (1)

H_2_O + *e^−^ → HO^●^ + H^●^ + *e^−^ (2)

O_2_ + O → O_3_ (3)

H_2_O + O →2 HO^●^ (4)

2 HO^●^ → H_2_O_2_ (5)

HO^●^ + O_3_ → HOO^●^ + O_2_ (6)

2 HOO^●^ → H_2_O_2_ + O_2._ (7)

**Figure 3:**
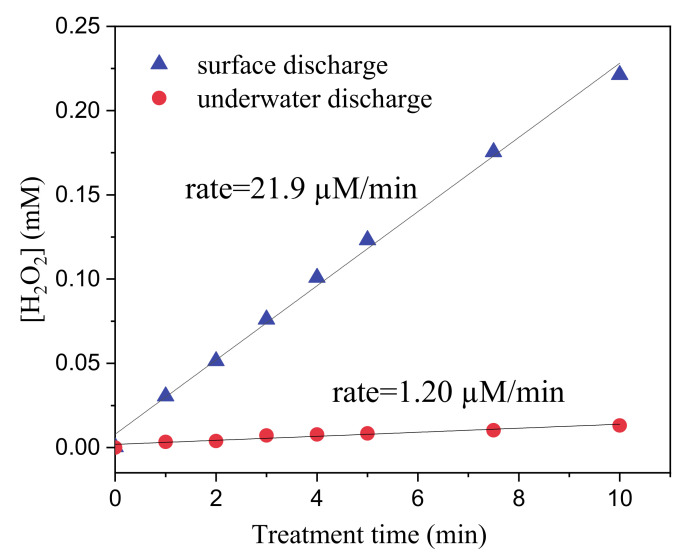
Concentration of hydrogen peroxide produced during plasma treatment of 50 mL of ultrapure water in PG reactor with surface and underwater discharge.

We quantified the formation of H_2_O_2_ and determined how the position of the discharge in the PG reactor could affect this production rate. 50 mL of MilliQ water were exposed to the two types of discharge for 10 min and the concentration of H_2_O_2_ was determined along treatment. Figure 3 shows the concentration of H_2_O_2_ as a function of the treatment time in the case of surface and underwater discharge. The results not only confirmed that H_2_O_2_ was formed along plasma treatment but also showed that the rate of formation is strongly dependent by the type of plasma discharge. Surface discharge led to a production rate 18-fold higher than underwater discharge (21.9 µM/min against 1.2 µM/min, respectively). This difference could be explained considering that, in the case of surface discharge, reactions (1–6) could take place at the liquid-air interface where the concentration of dissolved oxygen reach its maximum [31].

#### 3.2.3. Ozone and NO_x_ Production

**Figure 4:**
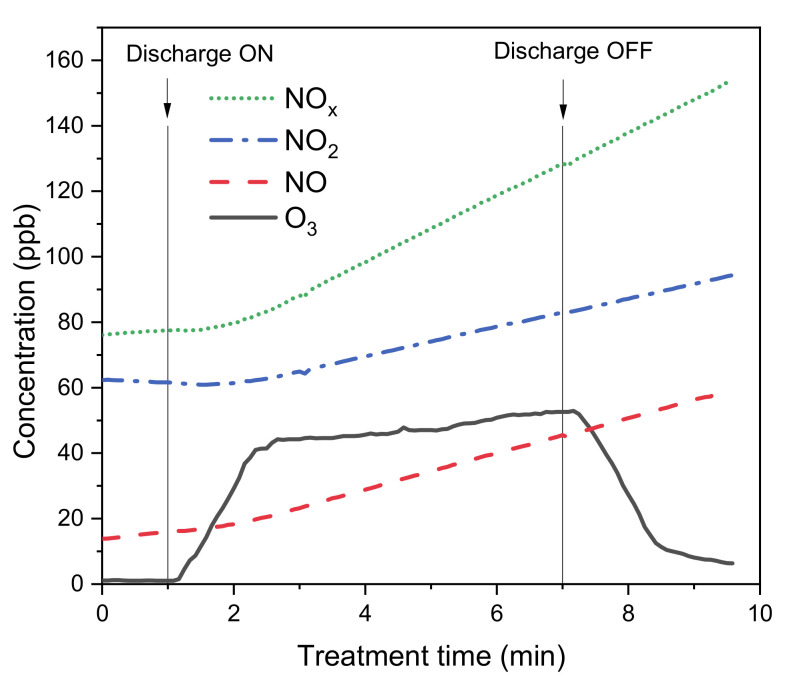
NO, NO_2_, NO_x_ and O_3_ concentration profiles measured before, during and after plasma treatment of 50 mL of ultrapure water in the PG reactor using superficial discharge.

The formation of ozone and NO_x_ in plasma discharges is commonly reported in the literature [31,32,33,34]. Significant concentration of O_3_ in the gas phase was measured when working with surface discharge. Data was registered for a total duration of 10 min divided in 1 min of background acquisition, 6 min of plasma discharge and 3 min of post-discharge analysis. NO, NO_2_, NO_x_ and O_3_ concentration profiles are shown in Figure 4. Background concentration of ozone in the room air was 1 ppb with negligible fluctuation over time. After plasma discharge was turned on, ozone concentration rapidly reached a stationary concentration of around 45 ppb and then dropped back to the pre-discharge values in around 4 min after interruption of the discharge. NO and NO_2_ concentrations on the other hand linearly increased throughout sampling period even after the discharge was turned off without reaching a stationary value. In particular, NO concentration increased at a slightly higher rate starting immediately after the plasma discharged was turned on and according to the literature it was the main NO_x_ specie formed inside the plasma: NO was oxidized by the ozone produced during the plasma treatment to give NO_2_ and some authors even reported this reaction to be quantitative [34]. The increase of the concentration of NO_2_ was concomitant with the change in the accumulation rate of ozone, as predicted by the reaction between NO and O_3_ that give NO_2_ and limited the accumulation of ozone. According to this theory, the production rate of both NO and O_3_ could be significantly higher than the measured ones.

### 3.3. Total Nitrogen and Nitrogen Speciation in Solution

**Figure 5:**
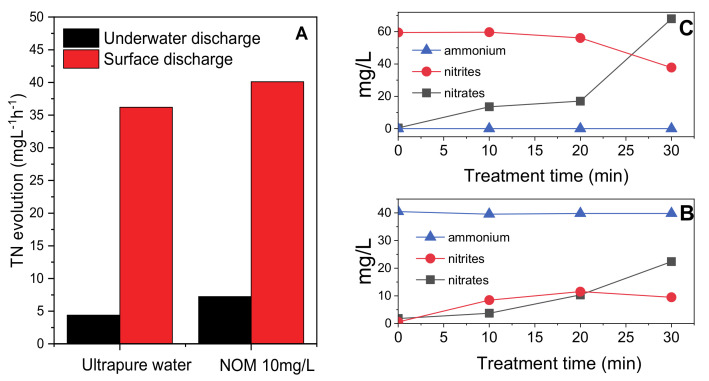
Comparison between the TN accumulation rate during treatment with surface and underwater discharge for a solution of ultrapure water and for a solution of NOM 10 mg/L (**A**); Concentration of ammonium, nitrite and nitrate ions over 30 min treatment with surface discharge in a solution on ammonium 40 mg/L (**B**) and in a solution of nitrite 60 mg/L (**C**).

As the formation of NO_x_ was observed in the gas phase, TN concentration with superficial and surface discharge was measured during experiments carried out in ultrapure water. In both cases the treatment duration was set to 8 min and TN was measured with intervals of 2 min. The nitrogen fixation appeared to be strongly dependent on the type of discharge adopted with TN increasing around 8 times faster with surface discharge with respect to underwater discharge due to the hydrolysis of the NO_x_ produced when the plasma discharge takes place in contact with air [35,36]. To investigate TN evolution in a more complex mixture, the same experiment was repeated replacing ultrapure water with a solution of Natural Organic Matter (NOM) 10 mg/L. Treatment time was extended to 30 min. The presence of NOM did not significantly modify the evidences observed in ultrapure water and confirmed that nitrogen fixation was more efficient with surface discharge. The TN concentration increased linearly (i.e., rate of production constant) with the treatment time both in ultrapure water and in NOM solution and both with underwater and surface discharge. The TN accumulation rates for the two samples are reported in Figure 5A (measured concentrations are reported in Figure S2). The zero order kinetic for the fixation of nitrogen (d[TN]/dt = constant) is in agreement with the hypothesis that this was directly related to the production of NO_x_ at the liquid/air interface as a consequence of the high energetic discharges, the frequency of discharge was constant during the treatments.

Furthermore, for the sample of NOM treated with surface discharge, nitrite, nitrate and ammonium concentrations were measured, in addition to TN, using ionic chromatography. These results showed that most part of nitrogen (~90% *w*/*w* of nitrogen) was fixed in solution in the form of nitrate ions with little formation of nitrite ions (<10% *w*/*w* of nitrogen) and negligible production of ammonium.

To better understand if the observed nitrate ions were formed directly in the plasma discharge or if they derived from the oxidation of other inorganic nitrogen species, two experiments were carried out. The first with ammonium (40 mg/L), the second with nitrites (60 mg/L) in ultrapure water with superficial discharge. The concentration of nitrites, nitrates and ammonium was monitored along treatment. When ammonium was spiked in the treated solution its concentration did not change along treatment meaning that ammonium was not produced in the first place and that the plasma discharge was not capable of oxidizing it at a significant rate. When nitrites were spiked in solution on the other hand, their concentration at the end of the treatment was reduced to around 40% of the initial value. At the same time the increase of nitrates’ concentration was considerably higher than before confirming that the plasma was able to oxidize a significant portion of nitrite to nitrate ions. Nitrates formation in the system under study takes therefore place both in a direct (especially in the first 20 min of treatment) and indirect way via the oxidation of previously formed nitrite ions (significant after 20 min of treatment). 

### 3.4. Metals Release from the Electrodes

Since the electrodes were visibly consuming with use, the concentrations of Fe, Cr, Ni, W and Hg was measured in sample of ultrapure water treated with surface discharge for 15 min. Iron and tungsten release was found to be significant being these two metals the main components of the electrodes used in this work (stainless steel and sintered tungsten). The release of chromium and nickel in treated solutions was limited while no mercury was detected (Table 1). The metal release in solution due to electrodes consumption is a potential drawback of this technology very little mentioned in the literature. These data highlight how an appropriate choice of electrodes material is crucial when designing water plasma applications. As an example, in the tested experimental conditions the release of iron was not a problem being its concentration lower than the common limit of law for the discharge of treated water in surface waters (e.g., for the Italian legislation 2 mg/L [37]) furthermore iron species in solution could trigger additional processes (e.g., Fenton and photo-Fenton ones) able to produce further reactive species. On the contrary the concentration of tungsten should be a significant problem, being its measured concentration >1 mg/L after 15 min of treatment.

### 3.5. Degradation of PFAS

Individual solutions of PFOA, PFOS and PFHxA were prepared in MilliQ and in groundwater matrices (chemical parameters of groundwater are reported in Table S3) at a concentration of 1 ppb. 50 mL of each sample solution were treated with surface plasma discharge in the PG reactor for 30 min and samples were taken at different time intervals during treatment in order to evaluate the degradation profiles. A mixture solution of these three compounds was prepared as well, in the same matrices with a final concentration of 1 ppb for each compound. Treatment time was extended to 1 h. All experiments were performed in triplicates.

In all the cases an exponential decay of the concentration of the substrates with the treatment time was observed (i.e., the degradation process had always a pseudo first order kinetics).

**Figure 6:**
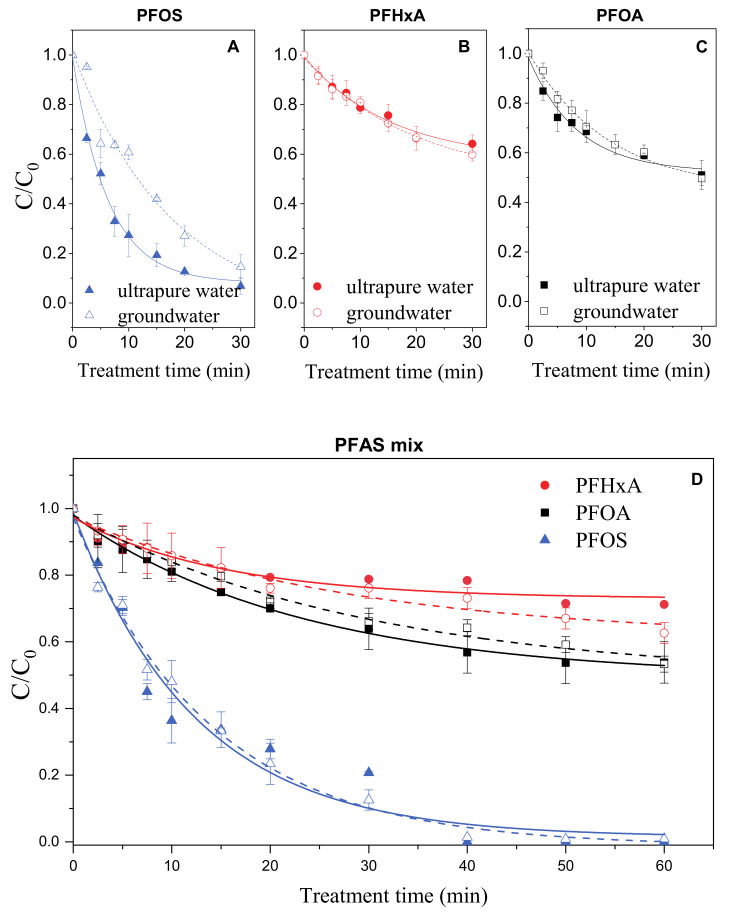
Degradation of pefluorooctanesulfonic acid (PFOS) (**A**), perfluorohexanoic acid (PFHxA) (**B**) and perfluorooctanoic acid (PFOA) (**C**) treated individually in ultrapure and groundwater matrices. Degradation of perfluoroalkyl substances (PFAS) treated in mixture (**D**) in ultrapure (solid symbols, solid lines) and groundwater (open symbols, dashed lines).

When compounds were treated individually, the best results were obtained for PFOS whose degradation was complete in ultrapure water and reached 85% in groundwater (Figure 6A). Removal efficiency of PFHxA on the other hand was slightly better in groundwater than in ultrapure water with degradation after 30 min of 40% and 35%, respectively (Figure 6B). PFOA was degraded of around 50% of initial concentration regardless the matrix (Figure 6C).

When treated in mixture the degradation profiles obtained for the three compounds showed that degradation is generally slower due to the competition among substrates for the produced reactive species. In mixture the data largely confirmed the results obtained for individual solutions with PFOS no longer detected after 30 min, PFOA removed for around 46% after 1 h in both matrices and PFHxA being the most recalcitrant compound with 29% removal in ultrapure water and 38% removal obtained in groundwater (Figure 6D). The first order degradation rate constants for the three investigated substrates both individually and in mixtures are reported in the Figure S3.

## 4. Conclusions

In this work, the operational parameters of a custom-built non-thermal plasma generator have been optimized using an experimental design. The analysis of the reactor gas phase revealed that significant production of ozone and NO_x_ takes place during water treatment. In solution NO_x_ hydrolysis led to an increase of total nitrogen (up to 40 mgL^−1^ h^−1^) mainly under the form of nitrate ions. A significant release of iron and tungsten in solution was observed as a consequence of the gradual oxidation of the electrodes material.

Secondly, the plasma generator running on optimized conditions has been used for the degradation of three perfluoroalkyl compounds PFOA, PFOS and PFHxA in solution. The compounds were treated both individually and in mixture using two different water matrices, ultrapure water and groundwater. In both cases PFOS removal was found to be the most efficient with the parent compound no longer detected after 30 min of treatment. The promising degradation results obtained for tested compounds and in particular for PFAS, suggest that this technology could be efficiently used for the treatment of contaminated groundwater, especially when water conductivity is low; environmental concentrations of PFAS in contaminated groundwater where the illicit discharge was done, typically fall within the concentration range explored in this work [9,38,39,40,41,42]. Being the PFAS probably the most recalcitrant compounds among the contaminants of emerging concern, the promising results with PFAS makes us confident that the plasma technology here investigated is also able to degrade less inert compounds. 

Preliminary investigations regarding the nature of the transformation products obtained during the plasma treatment of the tested perfluorinated compounds highlighted the formation of shorter-chain PFAS compounds in both ultrapure and groundwater. The identification and quantification of the transformation products of PFOA, PFOS and PFHxA produced during plasma treatment is currently under investigation.

## Figures and Tables

**Table 1:** Concentration in µg/L of Fe, Cr, Ni, W and Hg measured in a sample of ultrapure water treated with surface plasma discharge for 15 min. LOD = Limit of Detection.

min	Fe	Cr	Ni	W	Hg
0	4.8	0.0	3.5	142	<LOD
1	6.2	0.0	4.5	217	<LOD
3	5.3	0.0	4.6	285	<LOD
5	10.8	0.1	4.3	418	<LOD
10	25.2	1.7	5.3	787	<LOD
15	52.1	4.5	6.9	1118	<LOD

## Supplementary Materials

Method for the determination of H_2_O_2_ is reported at p. 1 of Supplementary Material, Figure S1: effect of applied polarity on the degradation of methyl orange, Figure S2: TN values measured in samples of ultrapure water (A) and for a solution of NOM 10 mg/L (B) treated with surface and underground discharge, Figure S3: First order kinetic constant of PFAS degradation (min^−1^) for individual treatment and in mixture in both ultrapure and underground water, Table S1: Experiments list of the DOE including the variables values used for each experiment and the observed experimental result (*k*), Table S2: Regression equation and *p* values obtained for the three parameters (frequency of discharge, electrodes distance and water conductivity) explored in the DOE, Table S3: Chemical parameters of underground water sampled in the city of Volpiano (Turin, Italy) used for the preparation of PFAS solutions.
