# Supplementary material for: PFAS Degradation in Ultrapure and Groundwater Using Non-Thermal Plasma
Source: Molecules. 2021 Feb 9;26(4):924. doi: 10.3390/molecules26040924 (PMC7916234; doi:10.3390/molecules26040924)
Supplement: Supplementary file 1 [file molecules-26-00924-s001.pdf]

PFAS degradation in ultrapure and groundwater using non-thermal plasma

Davide Palma [a], Dimitra Papagiannaki [b], Manuel Lai [c], Rita Binetti [b], Mohamad Sleiman [a], Marco Minella [d,*], Claire Richard [a,*]

[a] Université Clermont Auvergne, CNRS, Sigma Clermont, ICCF, 63178, Aubière, France

[b] SMAT S.p.A., Research Centre, C.so Unità d'Italia 235/3, 10127, Torino, Italy

[c] IRIS s.r.l., Via Papa Giovanni Paolo Secondo 26, 10043 Orbassano, Italy.

[d] Department of Chemistry and Interdepartmental Centre Nanostructured Interfaces and Surfaces (NIS), University of Torino, Via Pietro Giuria 5, 10125, Torino, Italy.

[*] Corresponding authors. E-mail addresses: marco.minella@unito.it, claire.richard@uca.fr

Supplementary Material

Measurement of hydrogen peroxide

The formation of H_2O_2 along plasma treatment was determined using two reagent solutions prepared as follows in two 250 mL flasks: solution A contained 16.5 g of potassium iodide, 0.5 g of sodium hydroxide and 0.05 g of ammonium heptamolybdate tetrahydrate, and solution B contained 10 g of potassium hydrogen phthalate. Both flasks were brought to volume using ultrapure water and the solutions were maintained under stirring until complete solubilisation. Determination of H_2O_2 was performed by mixing 3 mL of sample with 1 mL of solution A and 1 mL of solution B that were let to react for 1 min before measuring the absorbance at 350 nm. H_2O_2 concentrations were determined using a calibration curve.

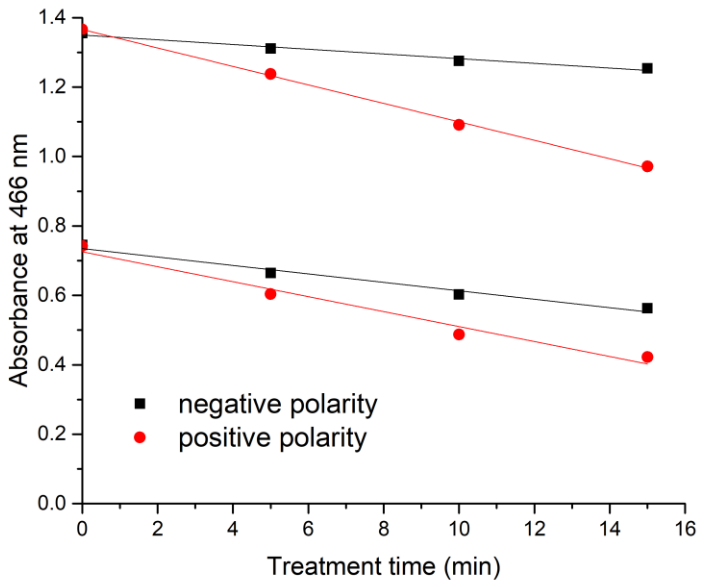

Figure S1. Degradation profile of MO 3×10^{-5} M (initial absorbance at 466 nm = 0.75) and 5.5×10^{-5} M (initial absorbance at 466 nm = 1.4) solutions treated using negative and positive polarity.

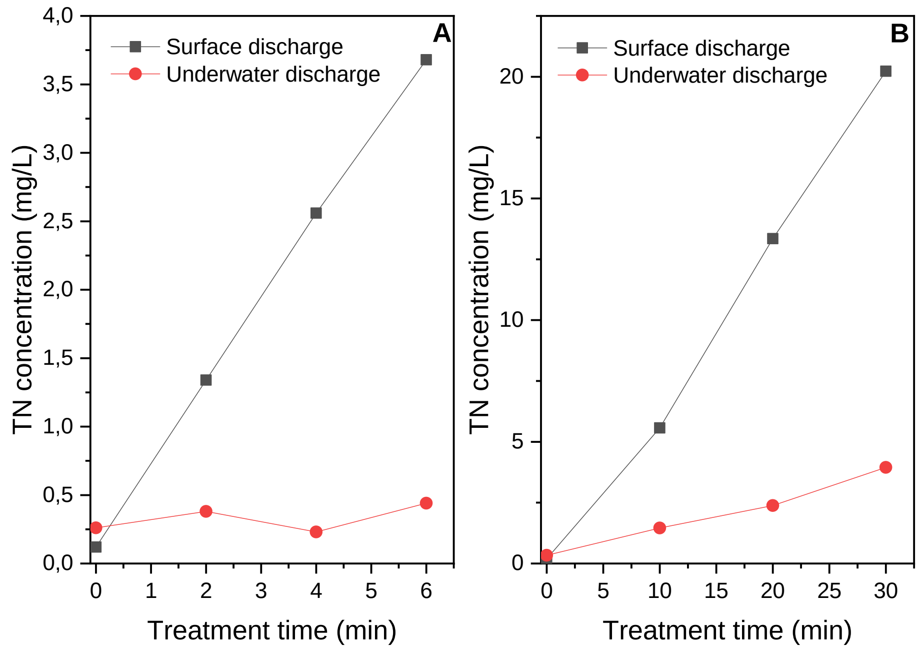

Figure S2. TN values measured in samples of ultrapure water (A) and for a solution of NOM 10 mg/L (B) treated with surface and underground discharge.

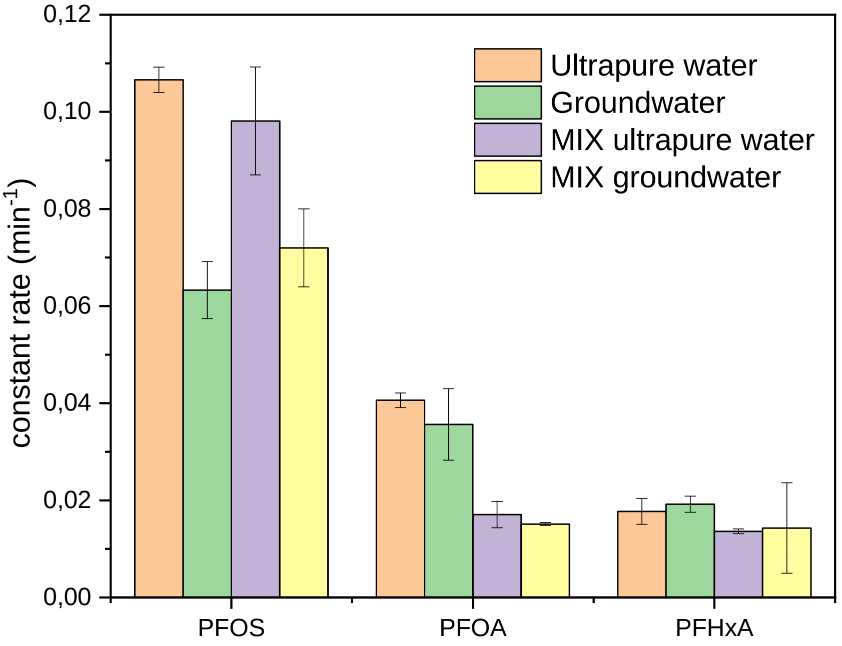

Figure S3. First order kinetic constant of PFAS degradation (min^{-1}) for individual treatment and in mixture in both ultrapure and underground water.

Table S1. Experiments list of the DOE including the variables values used for each experiment and the observed experimental result (*k*). Reported *k* values are average values of two repetitions.

Experiments number	Frequency (Hz)	Electr. Distance (mm)	H_2O conductivity (µS/cm)	*k*
1	5	1	20	0,2950
2	5	1	300	0,2937
3	5	10	20	0,2631
4	5	10	300	0,2297
5	17	1	20	0,6597
6	17	1	300	0,4042
7	17	10	20	0,7473
8	17	10	300	0,4448
9	5	5,5	160	0,1976
10	11	1	160	0,4415
11	11	5,5	20	0,3511
12	17	5,5	300	0,2900
13	11	5,5	160	0,3729

Table S2. Regression equation and *p* values obtained for the parameters (frequency of discharge, electrodes distance and water conductivity) explored in the DOE.

Parameter	Coeff.	Sigma	t	*p* value	Signif.	Contribution
Constant	1.883E-01	0.04245	4.437	0.0000	Very	
Frequency	3.144E-02	0.003184	9.874	0.0000	Very	32.30%
Electr. Distance	-7.976E-02	0.01107	-7.205	0.0000	Very	17.20%
H_20 conductivity	1.587E-03	0.000367	4.325	0.0004	Very	6.19%
Frequency × Electr. Distance	9.763E-04	0.000366	2.665	0.0158	Yes	2.35%
Frequency × H_20 conductivity	-8.206E-05	1.14E-05	-7.171	0.0000	Very	17%
Electr. Distance × Electr. Distance	6.383E-03	0.000948	6.734	0.0000	Very	15%
H_20 conductivity × H_20 conductivity	-3.847E-06	1.10E-06	-3.493	0.0026	Very	4.04%

Table S3. Chemical parameters of underground water sampled in the city of Volpiano (Turin, Italy) used for the preparation of PFAS solutions.

pH		8.1
Conductivity	µS/cm	195.45
TOC	µg/L	270
nitrites	mg/L	n.d.
nitrates	mg/L	8
Chlorides	mg/L	3
Sulphates	mg/L	8
Sodium	mg/L	4
Potassium	mg/L	1.0
Calcium	mg/L	22.8
Magnesium	mg/L	11.6
